# Enhancing Biochemical Resolution by Hyperdimensional Imaging Microscopy

**DOI:** 10.1016/j.bpj.2019.04.015

**Published:** 2019-04-22

**Authors:** Alessandro Esposito, Ashok R. Venkitaraman

**Affiliations:** 1 Medical Research Council Cancer Unit, University of Cambridge, Cambridge, United Kingdom

## Abstract

Two decades of fast-paced innovation have improved the spatial resolution of fluorescence microscopy to enable molecular resolution with low invasiveness and high specificity. Fluorescence microscopy also enables scientists and clinicians to map and quantitate the physicochemical properties (e.g., analyte concentration, enzymatic activities, and protein-protein interactions) of biological samples. But the biochemical resolving power of fluorescence microscopy is not as well optimized as its spatial resolution. Current techniques typically observe only the individual properties of fluorescence, thus limiting the opportunities for sensing and multiplexing. Here, we demonstrate a new, to our knowledge, imaging paradigm, hyperdimensional imaging microscopy, which quantifies simultaneously and efficiently all the properties of fluorescence emission (excited-state lifetime, polarization, and spectra) in biological samples, transcending existing limitations. Such simultaneous detection of fluorescence features maximizes the biochemical resolving power of fluorescence microscopy, thereby providing the means to enhance sensing capabilities and enable heavily multiplexed assays. Just as multidimensional separation in mass-spectroscopy and multidimensional spectra in NMR have empowered proteomics and structural biology, we envisage that hyperdimensional imaging microscopy spectra of unprecedented dimensionality will catalyze advances in systems biology and medical diagnostics.

## Introduction

The biochemical environment of tissues and cells can be probed either by analyzing the fluorescence of several naturally occurring, often metabolic-related, biomolecules (e.g., various forms of nicotinamide adenine dinucleotide in its oxidized or reduced forms NAD^+^/NADH) and flavin adenine dinucleotide (1, 2) or by analyzing the fluorescence of environmentally sensitive fluorophores (e.g., organic molecules and fluorescent proteins sensitive to pH) introduced into the sample by chemical or genetic means (3, 4). FRET (Förster resonance energy transfer) is also a well-established and widely used technique that enables cellular metabolism (e.g., with glucose and ATP probes (5, 6)) and signaling (e.g., with phosphorylation, acetylation, and methylation probes (7)) to be mapped on single living cells. Often, these assays alter several properties of fluorescence. For instance, the heterogeneous biochemical milieu of tissues introduces complex optical-biochemical signatures into a specimen’s fluorescence, or FRET alters the spectra, lifetime, and polarization of the FRET pair emission. However, state-of-the-art biochemical imaging techniques often rely just on the detection of simple optical features.

We hypothesized that the simultaneous detection of multiple characteristics of fluorescence would permit us to extend significantly the biochemical resolving power in fluorescence microscopy, thus supporting more precise measurements or increased multiplexing capabilities (e.g., multiple diagnostic markers or biochemical probes). Here, we illustrate the implementation of a novel, to our knowledge, detection paradigm that enables the parallel detection of all properties of fluorescence (“hyperdimensional detection;” see Supporting Materials and Methods, Text S1) and provide the first analytical tools (HDIM-toolbox; see Supporting Materials and Methods, Text S2) to handle such complex data sets. We demonstrate how hyperdimensional imaging microscopy (HDIM) maximizes the biochemical resolving power of fluorescence microscopy and provide proof-of-concept experiments to illustrate how its increased resolution and multiplexed capabilities could be utilized in biomedical and clinical applications.

## Materials and Methods

### Microscopy and general principles of HDIM image analysis

**Figure 1:**
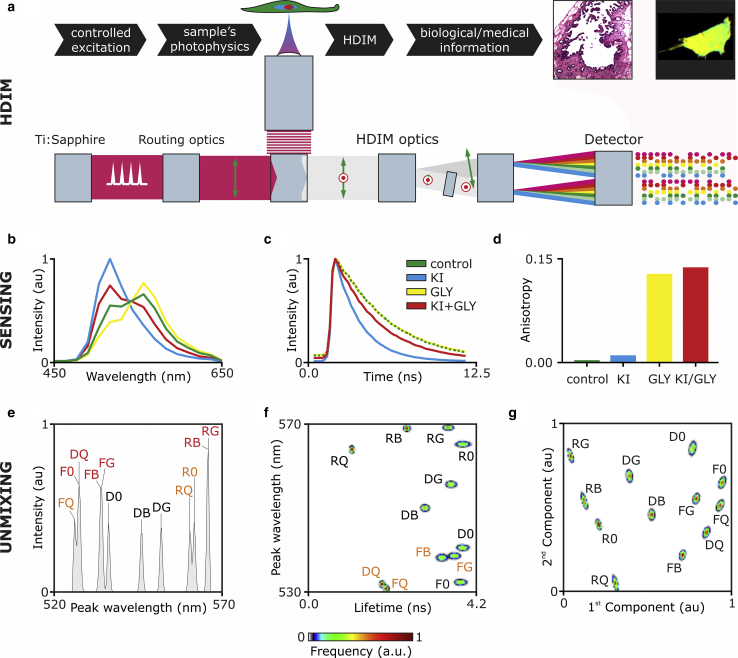
Sensing and unmixing by hyperdimensional imaging microscopy (HDIM). HDIM relies on the controlled excitation of the sample and by the analysis of biochemical signatures introduced into the photophysical properties of fluorophores within the sample (*a*). Sensing the biochemical environment of fluorophores (here R6G and FITC) can be achieved with the simultaneous detection of emission spectra (*b*), fluorescence lifetime (*c*), and anisotropy (*d*). Unmixing of different biochemical environment sensed by R6G and FITC is enhanced by the increased dimensionality of the analyzed photophysical signatures: spectral peak (*e*), spectral peak versus lifetime (*f*), and by PCA (*g*). Peaks are marked with the labels “F” for FITC, “R” for R6G, and “D” for an R6G and FITC mixture, followed by “0” (0% glycerol, 200 *μ*M KCl), G (65% glycerol, 200 *μ*M KCl), Q (0% glycerol, 100 *μ*M KCl, 100 *μ*M KI), or B (65% glycerol, 100 *μ*M KCl, 100*μ*M KI). Red and orange labels indicate nonresolved and partially overlapping peaks, respectively. The excitation is 840 nm.

Schematics of the microscope are shown in Supporting Materials and Methods (Fig. 1 *a*; Figs. S2 and S3). Briefly, two-photon excitation (TPE) is provided by a tunable femtosecond-pulsed Ti:sapphire laser (Chameleon Vision II; Coherent, Santa Clara, CA). TPE provides the ideal excitation for nanosecond lived excited-state lifetime estimation and a high dynamic range for anisotropy measurements (a maximum of 0.57 vs. 0.4 for one-photon excitation (8)); it also permits the simple separation of the infrared excitation light from ultraviolet-visible fluorescence emission spectra and second harmonic signals. Care should be taken to avoid instabilities of the polarization of the excitation light. The system we developed was built around a Leica SP5 confocal/multiphoton microscope (Leica Microsystems Ltd, Milton Keynes, UK), which uses a periscope formed by a polarization beam splitter (PBS) and a mirror. The PBS works together with a half-wave plate to finely tune the excitation power. The poor contrast ratio of a PBS and nonideal performance of the reflection utilized in the periscope introduced elliptical polarization at the entrance of the microscope, which we cleaned up with a Glan-Thompson polarizer (Newport, Irvine, CA; see Fig. S3). HDIM detection was achieved by coupling two grating-based spectrographs (200 nm bandwidth) with a PBS. Each spectrograph was equipped with a multianode photomultiplier tube and electronics for multidimensional time-correlated single-photon counting. All time-correlated single photon counting electronics, detectors, and spectrographs were purchased from Becker & Hickl (Berlin, Germany). Equipment for excitation, scanning, and detection is commercially available; hardware and software for the integration of these parts have been developed in-house. The system design is described in Figs. S2 and S3 and can be easily replicated. The HDIM-toolbox is freely available at a GitHub repository (http://www.github.com/alesposito/HDIM-toolbox) and described in Supporting Materials and Methods. At any given image pixel, this setup provides 16 spectral channels over 2 polarization states with arrival times typically histogrammed over 64 time bins. HDIM was calibrated with a laser comb provided by the standard laser lines of the confocal microscope (458, 488, 514, 561, 594, and 633 nm), back reflected by the objective lens and with a white-light-emitting diode with which light was scattered by a frosted glass. Typical acquisition times for images of 256 × 256 pixels were within 1–2 min (solutions or *Convallaria majalis* samples). Further details about sample preparation and imaging protocols can be found in Supporting Materials and Methods. Images were acquired with an HCX PL APO CS 40 × 1.25 NA oil objective with a 1.93 zoom, thus imaging a field of view of 200 *μ*m side.

### Sample preparation and analysis in solution

**Figure 2:**
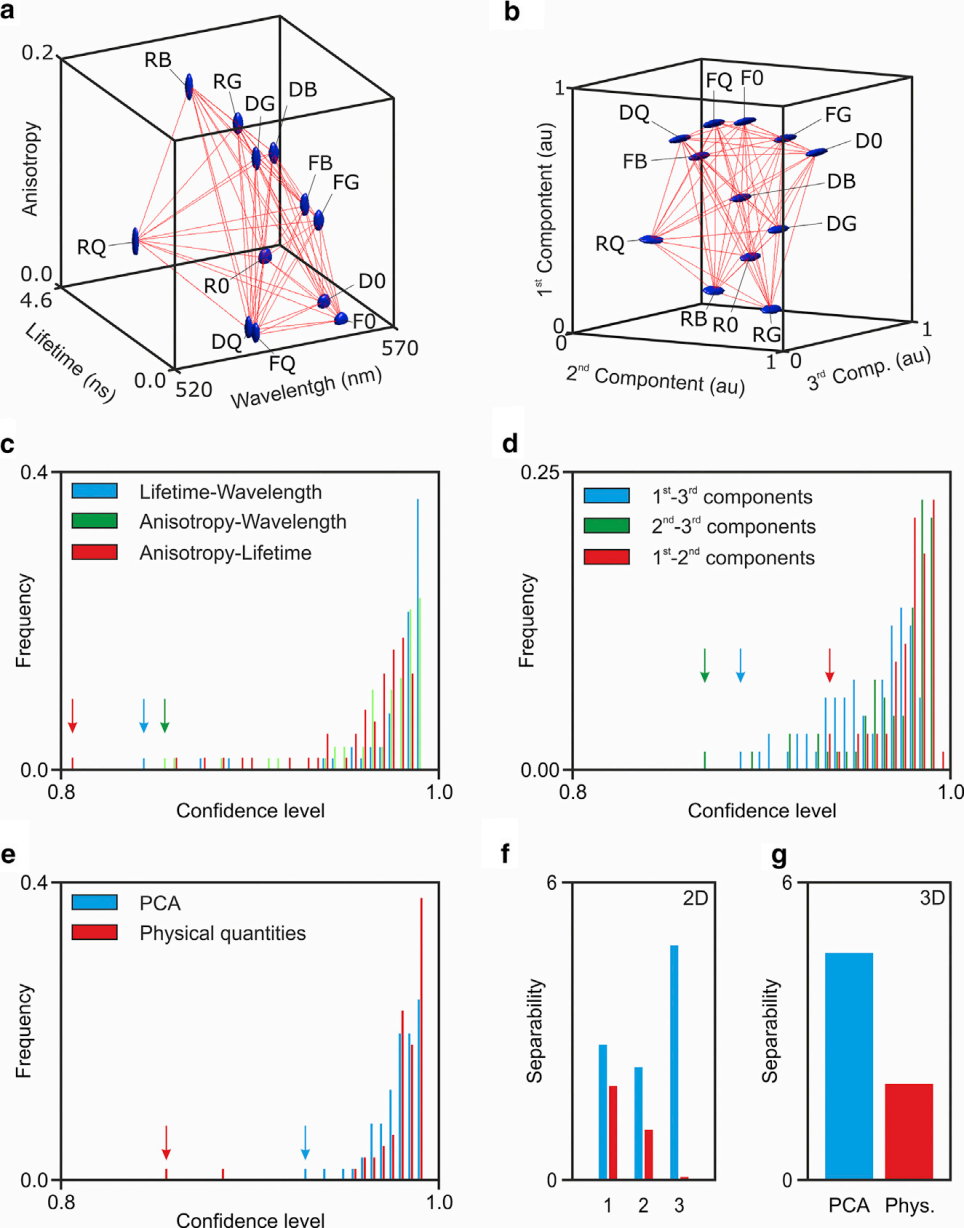
Improving biochemical resolving power by HDIM. We can build three-dimensional spectra utilizing physical properties of fluorescence ((*a*) lifetime versus anisotropy versus wavelength) or principal components (*b*). Each multidimensional spectrum (either two-dimensional as in Fig. 1 and Fig. S5 or three-dimensional in (*a*) and (*b*)) results in different statistical confidence for separating the tested samples. (*c*) shows how two-dimensional spectra of physical quantities provide good results but with several peaks that are less resolved. The arrows indicate the worst performances for each type of analysis. (*d*) Multivariate analysis improves significantly the confidence with which the closet objects can be separated; unsurprisingly, the first and second principal components (*red arrow*) provide the highest confidence. A direct comparison between PCA and physical quantities is shown for the three-dimensional spectra in (*e*). The arrows indicate the worst performance of the two methods also in this representation. (*f*) (*blue*: PCA spectra; *red*: physical quantities; one to three indicate the pairs as per *c* and *d*, from top to bottom). (*g*) A plot of the average separability index (*S*_*ij*_) as defined in the Supporting Materials and Methods is shown.

Fluorescent solutions were prepared from ethanol stock solutions of fluorescein isothiocyanate (FITC; 1 mM; Fluka Analytical, Sigma-Aldrich, St. Louis, MO) and rhodamine 6G (R6G, 5 mM; Sigma-Aldrich) to a final concentration of 10 and 1 *μ*M, respectively. Aqueous solutions were prepared with 200 *μ*M KCl (Sigma-Aldrich) and a constant ethanol concentration (10% v/v). Fluorophores were quenched by equimolar substitution of KCl for Kl (100 *μ*M; Sigma-Aldrich), and their rotational correlation time increased with 65% v/v glycerol (AnalaR NORMAPUR; VWR International Ltd, Lutterworth, UK). The concentrations of the fluorophores were selected to provide a similar brightness with TPE at 840 nm. We prepared 12 different solutions. We prepared three solutions in 200 *μ*M KCl: FITC (F), R6G (R), FITC + R6G (D); in 100 *μ*M KCl and 100 *μ*M Kl: FITC (FQ), R6G (RQ), FITC + R6G (DQ); in 200 *μ*M KCl and glycerol: FITC (FG), R6G (RG), FITC + R6G (DG); in 100 *μ*M KCl, 100 *μ*M Kl and glycerol: FITC (FB), R6G (RB), FITC + R6G (DB). Solutions were imaged sequentially in a glass-bottom microtiter plate. The characteristic spectra, fluorescence lifetimes, and polarization anisotropies displayed in Fig. 1, *b*–*d* and in Fig. S4 are shown after summing all photon counts of the HDIM data sets along all the dimensions, but the spectroscopic dimension of interest. For example, one-dimensional spectra were computed by summing the HDIM images over the *x*, *y*, and pairs of time- polarization or spectral- bins. The distributions shown in Fig. 1, *e*–*f* and Fig. S5, *a*–*f* were obtained by summing the HDIM images along pairs of spectroscopic features as well, but not over space. A single spectral feature (spectral peak, average fluorescence lifetime, and polarization anisotropy) was then evaluated in each pixel of the images, and the resulting values were histogrammed to display the variability of the measurements, with the resulting distributions summed to create a single plot showing their relative separations. Principal component analysis (PCA) (9) was performed on the full pixel ensemble of 12 HDIM data sets representative of the measured solutions after a 4 × 4 binning procedure in the *x* and *y* spatial dimensions for handling the large data sets. The trained PCA transform was then applied to the original full-size data set independently, and the distribution of individual components were then pooled together to show separations (Fig. 1 *g*, Fig. S4, *g*–*l*, and Fig. 2 *b*) as for the physical quantities (Fig. 1, *e*–*f*, Fig. 2 *a*, and Fig. S4, *a*–*f*) already described.

### Sample preparation and analysis of *C. majalis*

C*. majalis* sections stained with Safranin and Fast Green and then mounted were purchased from Leica Microsystems UK (category number: As3211; Milton Keynes, United Kingdom).

To provide digital images resembling counterstains used typically in histopathology (HE and 3,3′-diaminobenzidine (DAB)), we initially perform PCA on the pixel ensemble of an HDIM image. By definition, the principal component of PCA maximizes contrast among the pixel, hence providing a color channel with the maximal contrast within the acquired image. The subsequent components provide a decreasing level of contrast until they contain just noise. The digital stains are generated with the function “if_hdim_pca_rgb2dab” included in the HDIM-toolbox. Briefly, each of the first four PCA component (*n* = 1, 2, 3, 4) at each pixel location (*i*, *j*), was first stretched between the extreme values, PCA(*i*, *j*)′_*n*_ = (PCA(*i*, *j*)_*n*_−PCA^min^_*n*_)/(*s*(PCA^max^_*n*_−PCA^min^_n_)). PCA^min^_*n*_ is the minimum of each component within an image. However, aiming to moderate noise-dependent variations, PCA^max^_*n*_ was computed empirically as the sum of the median of PCA(*i*, *j*)_*n*_ with three SDs. *s* is a value computed to reach the desired level of saturation of the images, which is optionally left equal to one or computed to restretch the PCA(*i*, *j*)′_*n*_ values to saturate during the visualization at the 95% percentiles. All these operations are designed to achieve a good level of robustness in the automatic visualization of the stains. The color projections are then computed on the resulting PCA(*i*, *j*)′_*n*_ values. The HE-like representations (labeled as “PCA > DAB3” by the HDIM-toolbox) visualize the first three PCA components as the magenta, cyan, and black components of a CMYK (magenta, cyan, yellow, and black) image composite, where the yellow channel was left empty. The CMYK image composite was then projected to the red-green-blue (RGB) color space for visualization. The DAB-like representation (labeled as PCA > DAB4 by the HDIM-toolbox) was computed in the same way but included the fourth (shown) or the sum of additional components (data not shown) as the yellow channel of the CMYK composite before the color transform to the RGB color space. These transforms permitted us to visualize the first three or four PCA components in color spaces similar to histopathological counterstains.

## Results

### Improving biochemical resolving power

The formal description of the Fisher information for a multichannel, multiparametric detection demonstrates the net increase in the biochemical resolving power that can be achieved theoretically (10). In Fig. S1 and Supplemental Materials and Methods, Text S2, we provide a brief description of the theory and a simple graphical interpretation of the theoretical results. The photon partitioning theorem (10) predicts that the detection of photons into an increasing number of distinct “detection channels” tends to maximize information from the optical-biochemical system under investigation. Here, we test this prediction experimentally, with the engineering and testing of a detection system employing multiple parallel detection channels, each with electronics that generate histograms containing information about the polarization, color, and arrival time of each detected photon at every position within the sample.

Fig. 1 *a* and Figs. S2 and S3 depict the experimental setup and the conceptual representation of HDIM. A pulsed laser (here, a Ti:sapphire laser for TPE) provides tightly controlled excitation light of known timing, polarization, and spectra, which is delivered to the sample with a laser scanning microscope. Fluorophores and the biochemical environment of the sample reshape the excitation signal, introducing complex optical signatures in the emitted fluorescence that are fully characterized by hyperdimensional detection, achieved with a pair of multiwavelength time-correlated single-photon-counting detectors (see Materials and Methods). The photophysics (biochemistry) of the sample is thus described by 2048 values (i.e., photon counts accumulated into 16 spectral bins) two polarization states, and 64 time gates within each pixel of the acquired image. With the use of HDIM-tailored analysis algorithms, it is then possible to retrieve biochemical signatures from the complete photophysical characterization of the sample.

To demonstrate the sensing and unmixing capabilities of HDIM, we imaged various solutions prepared with 1 *μ*M R6G and 10 *μ*M fluorescein. Glycerol was used to reduce the rotational freedom of the fluorophores, and equimolar substitution of potassium chloride with the quencher potassium iodide was employed to reduce their fluorescence lifetime (see Figs. S4 and S5 for the complete data set). Fig. 1, *b*–*d* shows how the emission spectra, lifetime, and anisotropy of a mixture of R6G and fluorescein are altered by their biochemical environment and how HDIM can sense these changes. By means of spectra of increasing dimensionality, HDIM can successfully separate different physicochemical environments (Fig. 1, *e* and *f*). Fig. S5, *a*–*f* further demonstrates that the 12 different mixtures of R6G, fluorescein, glycerol, and potassium iodide can be resolved only with spectra of higher dimensionality compared to typical one-dimensional spectral information. We also tested the benefits of implementing PCA as mean of contrast enhancement for the analysis of HDIM data sets (see Fig. 1 *g*; Fig. S5, *g*–*l*). Three-dimensional spectra generated by photophysical features (Fig. 2 *a*) or principal components (Fig. 2 *b*) further illustrate the capability to increase the separability of different photophysical/biochemical features with spectra of higher dimensionality. To analyze the enhanced resolving power at increasing dimensionality, we quantified the separability (11) of different pixel clusters with the Euclidian distance between their centroids divided by the root sum of their variance (Fig. 2, *c*–*g*; see Supporting Materials and Methods). The separability of the pairs of pixel-clusters might either improve or deteriorate with spectra of higher dimensionality. However, Fig. 2 shows that the separability of the closest pixel cluster (i.e., the worst-resolved pair) improves at higher dimensionalities. It is possible to enumerate tens of different spectroscopic features (12) that could be extracted from an HDIM data set aiming to improve the separability of these pixel clusters. Instead, we have implemented PCA to achieve a representation of the data that provide the smallest possible dimensionality together with the advantage of enhanced resolution provided by multidimensional data sets. Fig. 2 shows that PCA can further improve the separability between different pixels. This is possible because PCA conveys all meaningful variation of spectra across different samples into the first components. Taken together, these experimental results and the underlying theory demonstrates that HDIM (multichannel and multiparametric imaging more generally) enhances the capability to sense and resolve differences in the photophysical/biochemical environment of the sample in fluorescence microscopy.

### Contrast enhancement during postprocessing

**Figure 3:**
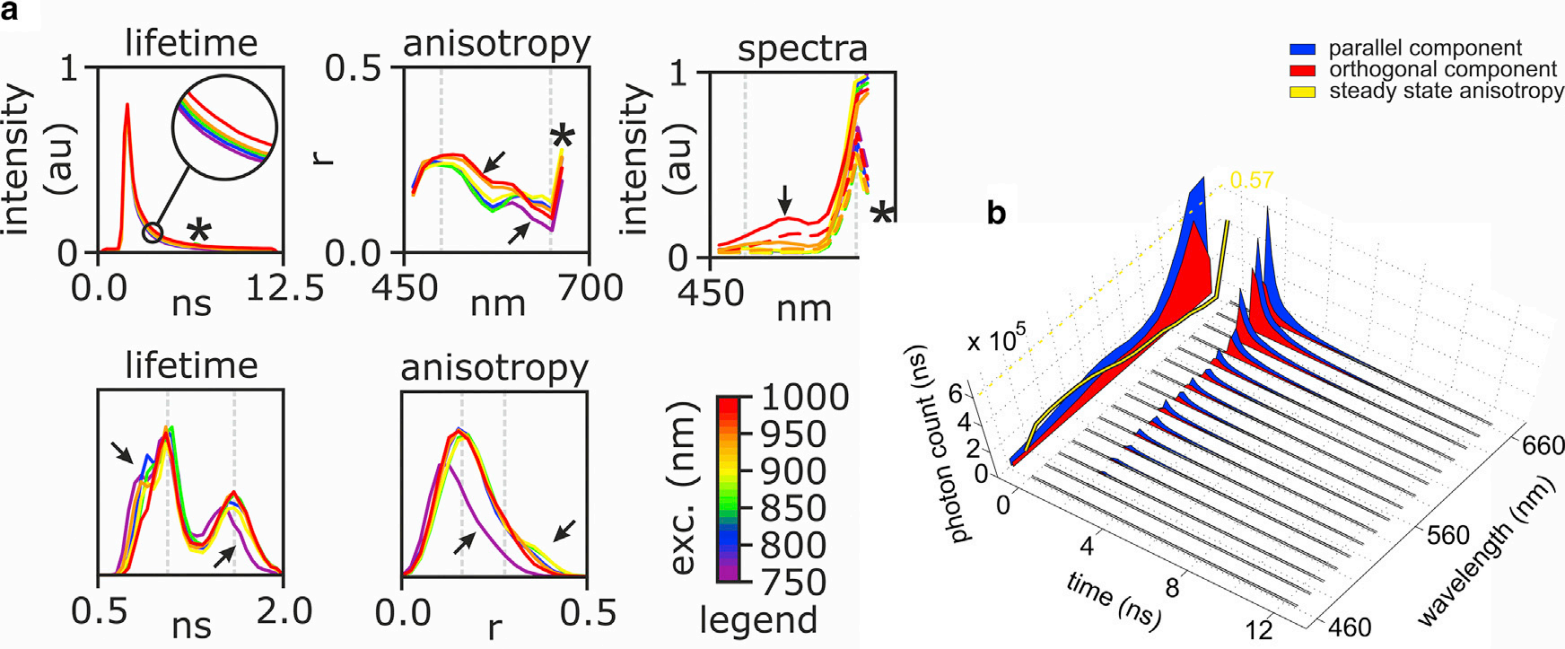
Sensing by hyperdimensional imaging microscopy (HDIM). (*a*) Fluorescence lifetime decays (*top left*), anisotropy spectra (*top center*), and emission spectra (*top right*) as a function of excitation wavelength measured on a single field of view of *C. majalis* are shown. The bottom panels show excitation-dependent distributions of fluorescence lifetimes (*bottom left*) and anisotropies (*bottom right*). The arrows and star highlight correlated features that are modulated by excitation wavelength. (*b*) An example of hyperdimensional spectral signature (HDSS) at 800 nm excitation wavelength is shown. Time decays are shown for its spectral and polarization components. On the back projection of the three-dimensional plot, polarization-dependent spectra and anisotropy spectrum are shown, with the dashed yellow line marking the maximum of fluorescence anisotropy of 0.57 that can be measured with TPE.

We then investigated whether the increased resolving power of HDIM could reveal structures, which would otherwise be invisible or poorly visible, when sensing individual optical properties. To test this possibility, we acquired images of *C. majalis* stained with Safranin and Fast Green, a typical sample used to test microscopy techniques. Fig. 3 illustrates the wealth of information that is acquired by HDIM. In this case, the data set is excitation resolved as well by scanning the Ti:sapphire laser from 750 to 1000 nm in steps of 50 nm. We demonstrate how fluorescence lifetime, anisotropy, and emission spectra change as a function of excitation wavelength (Fig. 3 *a*). The complexity of the optical signatures acquired by HDIM is shown in Fig. 3 *b* as hyperdimensional spectral signature (HDSS).

**Figure 4:**
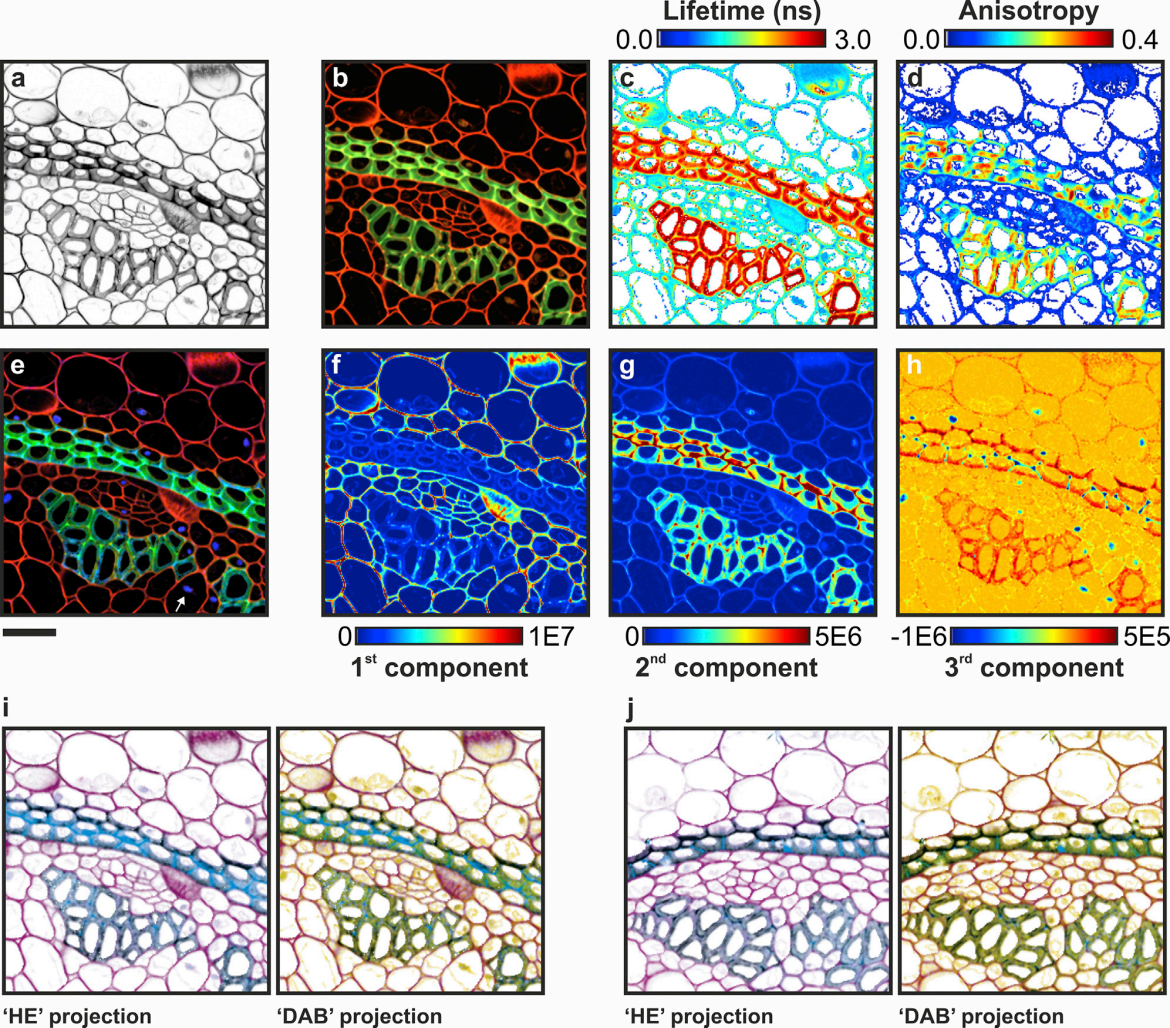
Unmixing by hyperdimensional imaging microscopy (HDIM). Images of *C. majalis*, shown as total photon counts (*a*), and projections of HDSS values as true color representation (*b*), fluorescence lifetime map (*c*), and fluorescence anisotropy map (*d*) are shown. Multivariate analysis of HDSS by PCA across the image provides a high-contrast RGB composite (*e*) obtained by the overlay of the first three principal components (*f*–*h*). Digital stains derived from PCA can be projected to a color space analogous to the HE and DAB counterstainings used in histopathology: (*i*) a field of view used for training PCA and (*j*) an independent imaged recolored with the same projection matrix are shown. Scale bar, 40 *μ*m. The excitation is 800 nm.

Fig. 4 *a* shows an intensity image of the sample excited at 800 nm. The specific optical properties of the sample can be mapped to two-dimensional maps through simple projections of the abstract 2048-multidimensional space where each pixel can be described. Projections can be either based on physical quantities (e.g., fluorescence anisotropy), statistical quantities (e.g., PCA or nonnegative matrix factorization) or perception-based features (e.g., true color). The latter is exemplified in Fig. 4 *b*, which shows an RGB composite image of the specimen as if it were observed through eyepieces by the naked eye. To achieve this representation, first the 2048-dimensional HDIM data set is projected on a spectral-only space, effectively summing all photons in each individual time- and polarization- bins. Subsequently, photons from each spectral bin is weighted accordingly to an eye-sensitivity matrix and summed up into three color channels. Similarly, representation of physical quantities can be synthesized by projecting the HDIM data on other dimensions without applying any weighing factor; for instance, Fig. 4, *c* and *d* shows synthetic fluorescence lifetime imaging microscopy (FLIM) and fluorescence anisotropy imaging microscopy (FAIM) images (see also Fig. S6) generated by projecting HDIM data sets onto the relevant dimensions. To assess if the increased photophysical/biochemical resolution of HDIM translates into contrast enhancement (Fig. 2 *b*), we also project the HDIM data set onto an RGB composite showing the first three principal components (Fig. 4, *e*–*h*; Fig. S6). PCA is agnostic about the composition of the sample, and it merely enhances the contrast for each and between each component. In fact, Fig. 4 *e* shows structures of *C. majalis* that color; FLIM and FAIM images do not highlight. From the inspection of the individual principal components (Fig. 4, *f*–*h*), it is possible to establish that Safranin and Fast Green are detected as first and second principal components, respectively. Autofluorescence is loaded into the third principal component thus providing an additional mean of contrast.

Fig. 4, *i* and *j* illustrates also how perception matrices can be exploited for possible future applications of HDIM to tissue diagnostics. Principal components can be projected to color spaces resembling typical counterstains used in histopathology, such as hematoxylin and eosin (HE; Fig. 4, *i* and *j*, *left panels*, two principal components) in addition to DAB-like stain (Fig. 4, *i* and *j*, *right panels*, three principal components). The full data set from which Fig. 4 *j* was computed is shown in Fig. S6 (see also HDIM-toolbox/if_hdim_pca_rgb2dab.m in Supporting Materials and Methods and Materials and Methods).

Taken together, these observations demonstrate that HDIM provides unprecedented sensing capabilities that can be exploited for contrast enhancement and to better resolve distinct biochemical/photophysical environments.

## Discussion

Previously, we introduced a generalized concept of resolution in fluorescence microscopy, through which we demonstrated that from a theoretical perspective, it is possible to increase information content by increasing the number of independent detection channels, thus enhancing the sensing and unmixing capabilities of fluorescence microscopy (see the photon partitioning theorem and its corollaries in (10), Fig. S1 and Supplemental Materials and Methods, Text S2). From this theoretical foundation, we hypothesized that the simultaneous detection of orthogonal properties of light, such as fluorescence lifetime, polarization, and spectra, could maximize the biochemical resolving power of fluorescence microscopy.

We report in this article the results of work that provide proof of concept for this hypothesis. We introduce here a new detection paradigm (HDIM), wherein a time-resolved spectropolarimeter built with off-the-shelf components is utilized to fully resolve the fluorescence emission of specimens. We present here the first experimental evidence that HDIM can, in the first instance, provide unprecedented sensing capabilities. Rather than using filters or analyzers to generate images during acquisition, HDIM data sets store the full spectroscopic information of a specimen, resulting in data sets that can be projected onto spectral, lifetime, and polarization dimensions during postprocessing. We have also shown that projections can be performed not just on physical features but by using statistical tools and performing perception-based projections. We envisage that HDIM will be particularly valuable when the optical signatures of a biological phenomenon are unknown (e.g., in tumor imaging) in which HDIM sensing capabilities can be utilized as an artificial means of contrast enhancement.

Notably, our findings confirm that, as hypothesized, HDIM significantly enhances the resolving power in microscopy. HDIM exhibits spatial resolution equivalent to a laser scanning confocal or two-photon microscope; but in contrast, HDIM delivers enhanced resolving power to distinguish differences in photophysical properties. Therefore, HDIM can reveal structures that are photophysically distinct but that might be invisible to individual techniques (multicolor or hyperspectral imaging, FLIM, and FAIM). Therefore, the capability to better resolve distinct emitters should also increase the capability to multiplex a larger number of fluorophores with known characteristics.

The analogy of a microscope as an information channel can be instructive to better understand the results we have presented here. When we prepare a fluorescence-based assay, we encode one or more random variables of interest *x* (e.g., the concentration of analytes, rotational diffusions, or FRET efficiencies) into the spectroscopic features of the fluorophores. The transmitter (the light source and fluorophores) multiplexes information in the spectral, time, and polarization domains through the process of fluorescence. The HDSS of a sample (e.g., HDSS(*x*)) changes smoothly with *x*, and it is often highly correlated along different spectroscopic features. For instance, the change in relative abundance of two fluorophores exhibiting different fluorescence spectra, lifetime, and anisotropy will result in a highly correlated change across the time, spectral, and polarization domains of the HDSS. The receiver (the optics, detectors, and analysis algorithm) gather back information to reconstruct the message transmitted, *x*. By operating on the three orthogonal domains in which information is spread, HDIM is an efficient receiver capable to demultiplex most of the available information otherwise lost and to retrieve a more precise representation of the original message (*x*).

We have previously described that the addition of detection channels in fluorescence microscopy might result in practical disadvantages (10) (e.g., increased readout noise, optical losses, or cost). However, efficient dispersive optics provide very high collection efficiencies (>80%; see, for example, (13)); single-photon counting provides high-detection efficiencies limited only by intrinsic Poisson noise of photon detection with virtually no readout noise, and the typical speed limitations of photon-counting electronics are now overcome by recent developments (13, 14, 15). Furthermore, technologies currently beyond state-of-the-art, for instance, energy-resolving high-temperature superconducting single-photon detectors might provide efficient architectures for multiparametric detectors also in fluorescence microscopy (16) in the future. Therefore, both with the elimination of technological barriers and by improving our understanding of information theory in fluorescence detection, we can significantly improve the resolving power of fluorescence microscopy for the benefit of multiplexed sensing and biochemical imaging.

With faster and more cost-effective detection technologies being more readily available (13, 17, 18, 19), multiplexed detection technologies could be employed in a range of applications beyond the specialist laboratory. The advantages of multiparametric detection have been previously illustrated, ranging from applications in single-molecule spectroscopy (12) to fluorescence microscopy (20), including metabolic imaging (21, 22), quantification of interactions and molecular diffusion (23, 24), and tumor imaging (1, 25). A better understanding of a generalized concept of resolution in fluorescence microscopy and the implementation of technologies such as HDIM may thus impact several areas of biomedical relevance. By providing enhanced sensing and unmixing capabilities, HDIM may find utility, for instance, in contrast enhancement for label-free tissue imaging in maximizing the multiplexing capabilities of diagnostic markers in histopathology or fluorescent probes in living cells (26). Furthermore, although we report here on imaging applications, hyperdimensional detection can provide the same advantages when applied to spectroscopy or flow cytometry. To empower the development of such applications, we have shared the software “HDIM-toolbox,” a toolbox that might facilitate the development of advanced analytical tools by the broader community. We suggest that heavily multiplexed imaging applications will synergize with emerging technologies such as smart pixels and deep learning to significantly advance current capabilities for machine vision and a broad range of biomedical applications.

## Author Contributions

A.E. designed and executed the experiments, engineered the microscope and the analytical tools, and analyzed the data. A.R.V. contributed to the critical analysis of the results. A.E. and A.R.V. wrote the manuscript.

## Supporting Material

Document S1. Supporting Materials and Methods and Figs. S1–S6

Document S2. Article plus Supporting Material

## References

[1] Fereidouni, F., A. N. Bader, …, H. C. Gerritsen. 2014. Phasor analysis of multiphoton spectral images distinguishes autofluorescence components of in vivo human skin. J. Biophotonics. 7:589-596. 10.1002/jbio.201200244 23576407

[2] Trinh, A. L., H. Chen, …, Y. H. Zhou. 2017. Tracking functional tumor cell subpopulations of malignant glioma by phasor fluorescence lifetime imaging microscopy of NADH. Cancers (Basel). 9:168. 10.3390/cancers9120168 PMC5742816 29211022

[3] Miyawaki, A., J. Llopis, …, R. Y. Tsien. 1997. Fluorescent indicators for Ca2+ based on green fluorescent proteins and calmodulin. Nature. 388:882-887. 10.1038/42264 9278050

[4] Kawanishi, T., L. M. Blank, …, R. Y. Tsien. 1989. Ca2+ oscillations induced by hormonal stimulation of individual fura-2-loaded hepatocytes. J. Biol. Chem. 264:12859-12866. 2473983

[5] Kotera, I., T. Iwasaki, …, T. Nagai. 2010. Reversible dimerization of Aequorea victoria fluorescent proteins increases the dynamic range of FRET-based indicators. ACS Chem. Biol. 5:215-222. 10.1021/cb900263z 20047338

[6] Maioli, V., G. Chennell, …, C. Dunsby. 2016. Time-lapse 3-D measurements of a glucose biosensor in multicellular spheroids by light sheet fluorescence microscopy in commercial 96-well plates. Sci. Rep. 6:37777. 10.1038/srep37777 PMC5122899 27886235

[7] Rowland, C. E., C. W. Brown, …, J. B. Delehanty. 2015. Intracellular FRET-based probes: a review. Methods Appl. Fluoresc. 3:042006. 10.1088/2050-6120/3/4/042006 29148511

[8] Volkmer, A., V. Subramaniam, …, T. M. Jovin. 2000. One- and two-photon excited fluorescence lifetimes and anisotropy decays of green fluorescent proteins. Biophys. J. 78:1589-1598. 10.1016/S0006-3495(00)76711-7 PMC1300756 10692343

[9] Le Marois, A., S. Labouesse, …, R. Heintzmann. 2017. Noise-corrected principal component analysis of fluorescence lifetime imaging data. J. Biophotonics. 10:1124-1133. 10.1002/jbio.201600160 27943625

[10] Esposito, A., M. Popleteeva, and A. R. Venkitaraman. 2013. Maximizing the biochemical resolving power of fluorescence microscopy. PLoS One. 8:e77392. 10.1371/journal.pone.0077392 PMC3810478 24204821

[11] Kollner, M., and J. Wolfrum. 1992. How many photons are necessary for fluorescence-lifetime measurements. Chem. Phys. Lett. 200:199-204.

[12] Prummer, M., B. Sick, …, U. P. Wild. 2004. Multiparameter microscopy and spectroscopy for single-molecule analytics. Anal. Chem. 76:1633-1640. 10.1021/ac034976g 15018561

[13] Popleteeva, M., K. T. Haas, …, A. Esposito. 2015. Fast and simple spectral FLIM for biochemical and medical imaging. Opt. Express. 23:23511-23525. 10.1364/OE.23.023511 26368450

[14] Gersbach, M., R. Trimananda, …, E. Charbon. 2010. High frame-rate TCSPC-FLIM using a novel SPAD-based image sensor. In Proceedings Volume 7780, Detectors and Imaging Devices: Infrared, Focal Plane, Single Photon, E. L. Dereniak, J. P. Hartke, …, M. Razeghi, eds. (SPIE NanoScience + Engineering), p. 77801H.

[15] Krstajić, N., J. Levitt, …, R. Henderson. 2015. 256 × 2 SPAD line sensor for time resolved fluorescence spectroscopy. Opt. Express. 23:5653-5669. 10.1364/OE.23.005653 25836796

[16] Natarajan, C. M., M. G. Tanner, and R. H. Hadfield. 2012. Superconducting nanowire single-photon detectors: physics and applications. Supercond. Sci. Technol. 25:063001.

[17] Esposito, A., H. C. Gerritsen, …, F. S. Wouters. 2006. Innovating lifetime microscopy: a compact and simple tool for life sciences, screening, and diagnostics. J. Biomed. Opt. 11:34016. 10.1117/1.2208999 16822066

[18] Zhao, Q., B. Schelen, …, I. T. Young. 2012. Modulated electron-multiplied fluorescence lifetime imaging microscope: all-solid-state camera for fluorescence lifetime imaging. J. Biomed. Opt. 17:126020. 10.1117/1.jbo.17.12.126020 23323290

[19] Li, D. D. U., J. Arlt, …, R. K. Henderson. 2011. Video-rate fluorescence lifetime imaging camera with CMOS single-photon avalanche diode arrays and high-speed imaging algorithm. J. Biomed. Opt. 16:096012. 10.1117/1.3625288 21950926

[20] Bird, D. K., K. W. Eliceiri, …, J. G. White. 2004. Simultaneous two-photon spectral and lifetime fluorescence microscopy. Appl. Opt. 43:5173-5182. 10.1364/ao.43.005173 15473237

[21] Vishwasrao, H. D., A. A. Heikal, …, W. W. Webb. 2005. Conformational dependence of intracellular NADH on metabolic state revealed by associated fluorescence anisotropy. J. Biol. Chem. 280:25119-25126. 10.1074/jbc.M502475200 15863500

[22] Yu, Q., and A. A. Heikal. 2009. Two-photon autofluorescence dynamics imaging reveals sensitivity of intracellular NADH concentration and conformation to cell physiology at the single-cell level. J. Photochem. Photobiol. B. 95:46-57. 10.1016/j.jphotobiol.2008.12.010 PMC2739809 19179090

[23] Levitt, J. A., P. E. Morton, …, K. Suhling. 2015. Simultaneous FRAP, FLIM and FAIM for measurements of protein mobility and interaction in living cells. Biomed. Opt. Express. 6:3842-3854. 10.1364/BOE.6.003842 PMC4605044 26504635

[24] Nguyen, T. A., P. Sarkar, …, S. S. Vogel. 2012. Fluorescence polarization and fluctuation analysis monitors subunit proximity, stoichiometry, and protein complex hydrodynamics. PLoS One. 7:e38209. 10.1371/journal.pone.0038209 PMC3364239 22666486

[25] Fereidouni, F., K. Reitsma, and H. C. Gerritsen. 2013. High speed multispectral fluorescence lifetime imaging. Opt. Express. 21:11769-11782. 10.1364/OE.21.011769 23736399

[26] Fries, M. W., K. T. Haas, …, A. Esposito. 2018. Multiplexed biochemical imaging reveals caspase activation patterns underlying single cell fate. bioRxiv, doi: 10.1101/427237.

