# Supplementary material for: Enhancing Biochemical Resolution by Hyperdimensional Imaging Microscopy
Source: Biophys J. 2019 Apr 22;116(10):1815–22. doi: 10.1016/j.bpj.2019.04.015 (PMC6531829; doi:10.1016/j.bpj.2019.04.015)
Supplement: Document S1. Supporting Materials and Methods and Figs. S1–S6 [file mmc1.pdf]

Biophysical Journal, Volume *116*

Supplemental Information

Enhancing Biochemical Resolution by Hyperdimensional Imaging Microscopy

Alessandro Esposito and Ashok R. Venkitaraman

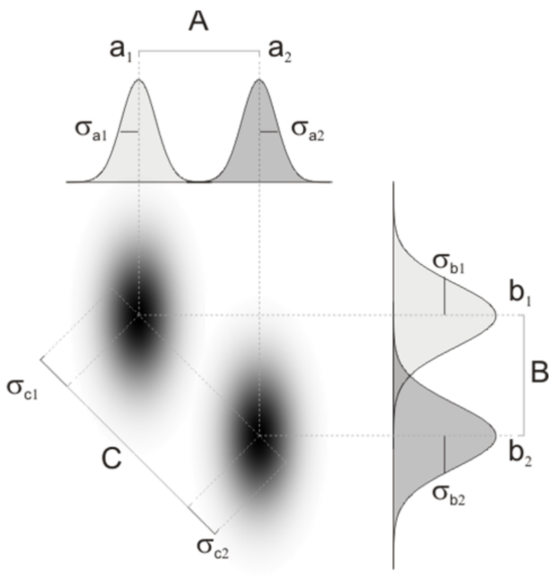

Figure S1 | Biochemical resolving power and multi-dimensional detection. A typical microscope measures one parameter (*e.g.*, fluorescence lifetime or anisotropy). *a* and *b* represent two features that discriminate between two biochemical environments. a_1, a_2, b_1 and b_2 are measured with uncertainties σ_{a1}, σ_{a2}, σ_{b1} and σ_{b2}, respectively. The resolving power of microscopes designed to measure either *a* or *b*, will be equal to $R_A = A (\sigma_{a1}^2 + \sigma_{a2}^2)^{-1/2}$ and $R_B = B (\sigma_{b1}^2 + \sigma_{b2}^2)^{-1/2}$, where A and B are the absolute differences between a_1, a_2 and b_1, b_2. If a and b can be measured at the same time, it will be possible to distinguish the two biochemical environments with a multi-dimensional feature *c* that provides increased resolving power $R_C = (R_A^2 + R_B^2)^{-1/2}$.

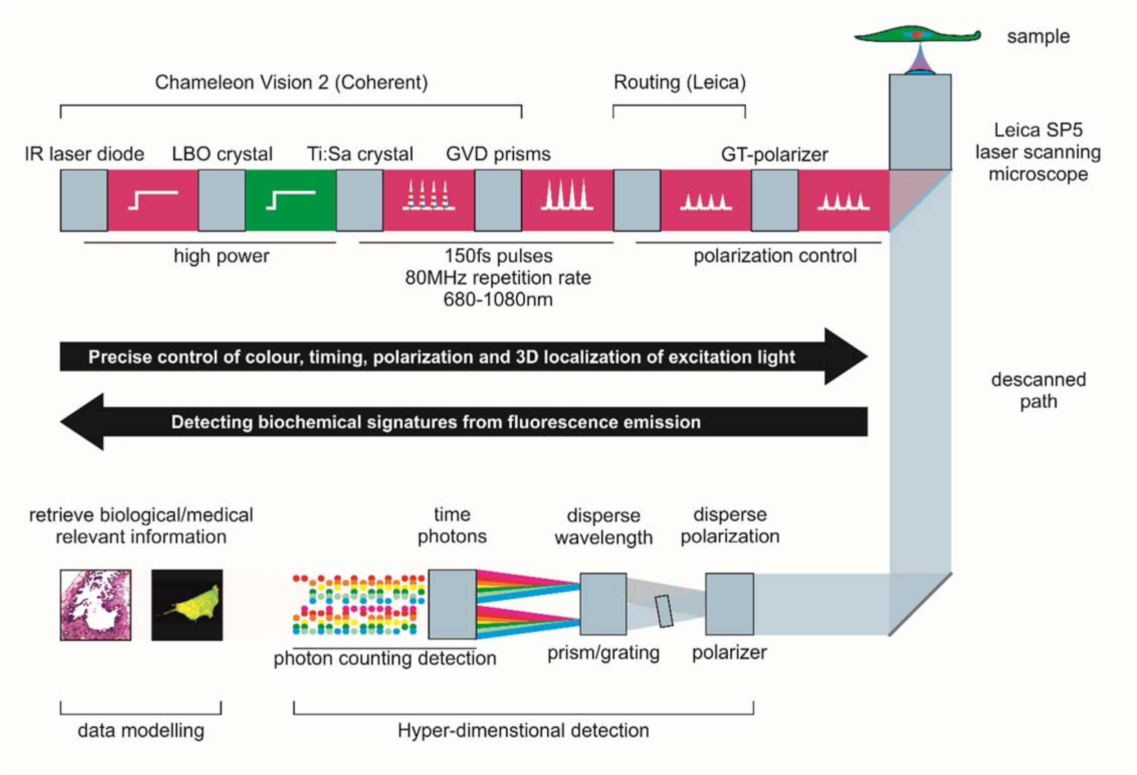

Figure S2 | HDIM vision and diagram. This first generation HDIM system is based on a Ti:Sapphire laser with group velocity dispersion compensation (Chameleon Vision 2, Coherent UK Ltd.) to provide well defined and tunable excitation light. Routing optics deliver the excitation light to a confocal laser scanning microscope (Leica SP5, Leica Microsystems UK, Ltd.) after the polarization of the excitation light was cleaned with a Glan polarizer. Fluorescence emission is then collected by the objective of the microscope, de-scanned and routed onto external in-house developed detectors. A polarizer beam splitter and a linear polarizer split fluorescence onto two spectrographs equipped with multi-anode PMTs and electronics for TCSPC (Becker&Hickl GmbH). Bespoke algorithms are then used to retrieve information of biological or biomedical relevance from complex HDIM datasets.

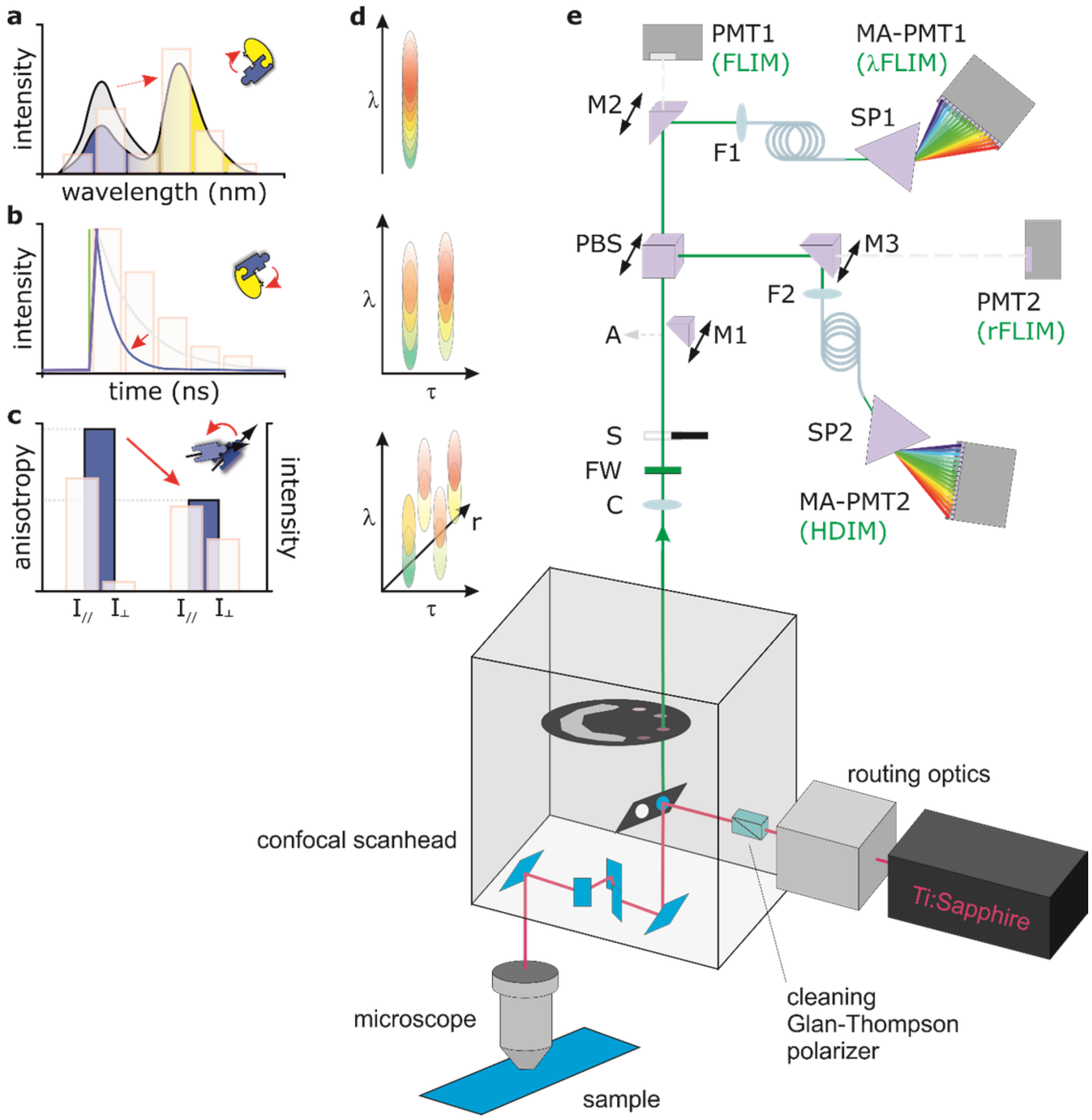

Figure S3 | Experimental setup. Conceptual representation of the sensing (**a-c**) and un-mixing (**d**) capabilities we aimed to achieve with HDIM. For instance, biochemistry can be interrogated by FRET-based probes by exploiting the FRET-dependent reduction of donor fluorophore quantum yield and sensitization of acceptor emission (**a**), the reduction of donor fluorescence lifetime (**b**) and the reduction of the acceptor fluorescence anisotropy (**c**). A number of spectral detection channels, time-gates and polarization-sensitive detection can be therefore exploited for both sensing (**a-c**) and un-mixing (**d**). The first generation of HDIM was not time resolved and have been already described (1). This second generation based on TCSPC and two-photon excitation is a completely new setup (**e**) and integrated in a multi-modal system that helped to characterize this first prototype. C, camera lens (AC254100A - Thorlabs); FW, filter-wheel (FW103/M - Thorlabs); S, shutter (9003-0212 – Becker&Hickl); A, auxiliary port; M1-3, turning mirrors (CM1-P01 - Thorlabs) controlled with linear servos (Firgelli L12-30-50-06-R by Active Robots); F1/2, light guide coupling lenses (AC254030A -

Thorlabs); PBS, polarization beam-splitter (CM1 PBS251 - Thorlabs); PMT1-2, (HPM-100-40 – Becker&Hickl); SP1-2, grating-based spectrographs (MS125 - Becker&Hickl); MA-PMT1-2, multi-anode photo-multiplier tubes and TCSPC electronics (PML-16-4-C and SPC152 – Becker&Hickl).

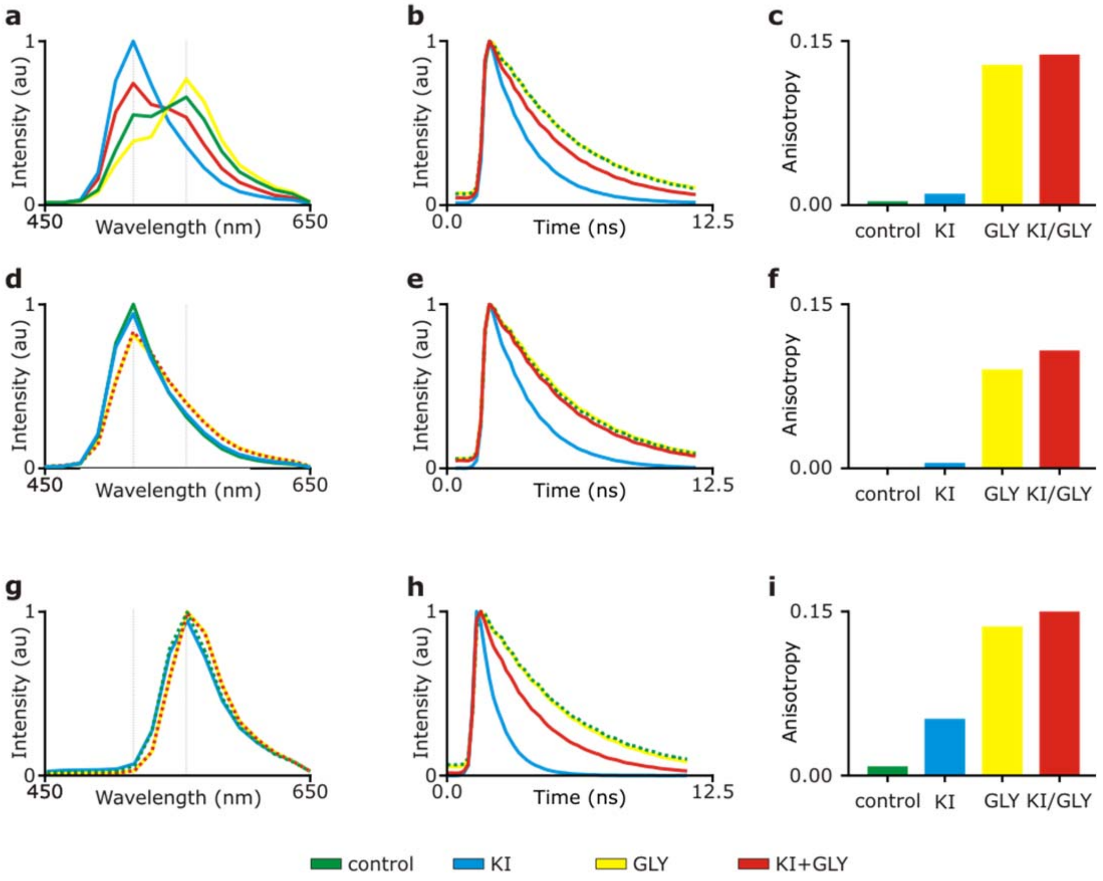

Figure S4 | Sensing by HDIM. A mixture of R6G (1µM) and FITC (10µM) was imaged in the various combinations without (control) or with 65% glycerol (GLY) or with equimolar substitution of potassium iodide (100 µM) for potassium chloride. The different effects of the quencher potassium iodide or glycerol on the rotation correlation time and quantum efficiency of the two fluorophores can be visualized by spectral (**a**, **d** and **g**), lifetime (**b**, **e** and **h**) and anisotropy (**c**, **f** and **i**) analysis of the HDIM datasets by summing all photon-counts along all the dimensions of the HDIM hyper-volume (x,y, time-, spectral- and polarization- bins) except the feature that is shown. Panels **a-c**, **d-f** and **g-i** shows the spectroscopic features for the mixture of FITC/R6G, FITC alone and R6G alone, respectively. The analysis for the mixture of the two fluorophores (**a-c**) is also shown as part of **Fig. 1.** As expected by the Perrin equation (2), correlation between fluorescence anisotropy and quenching are noticeable because of the reduction of fluorescence lifetimes.

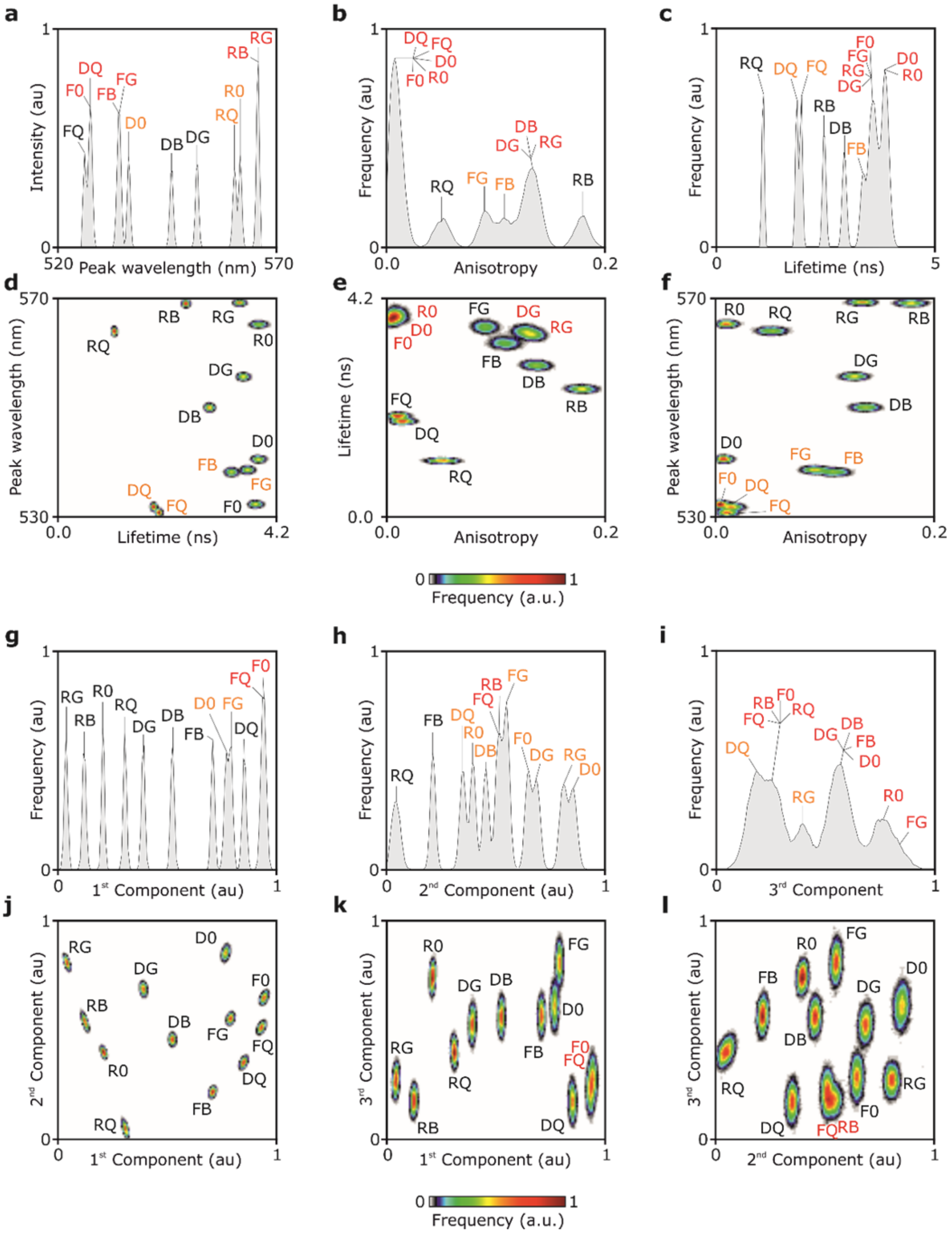

Figure S5 | Unmixing by HDIM. The frequency distributions of pixels with a given peak wavelength (**a**), fluorescence anisotropy (**b**) and fluorescence lifetime (**c**) values for the samples containing only FITC (F), Rhodamine 6G (R), the mixture of the two fluorophores (D) in 200 µM KCl (0), 100 µM KCl and 100 µM KI (Q), 65% Glycerol (G) or 100 µM KCl, 100 µM KI (Q), 65% Glycerol (B) shows ambiguous peak assignment to respective samples. In black we noted well resolved samples, in orange, not well separated peaks and red fully overlapping peaks. Bidimensional diagrams for peak wavelength *vs* lifetime (**d**), fluorescence lifetime *vs* polarization anisotropy (**e**), and peak wavelength *vs* anisotropy (**f**) improve separability, but not fully. Multi-dimensionality reduction algorithms like principal component

analysis increase the separability of the samples with each component alone (**g-f**), as two-dimensional histograms (**j-l**) or spectra of increasing dimensionality (**Figs. 1-2**)

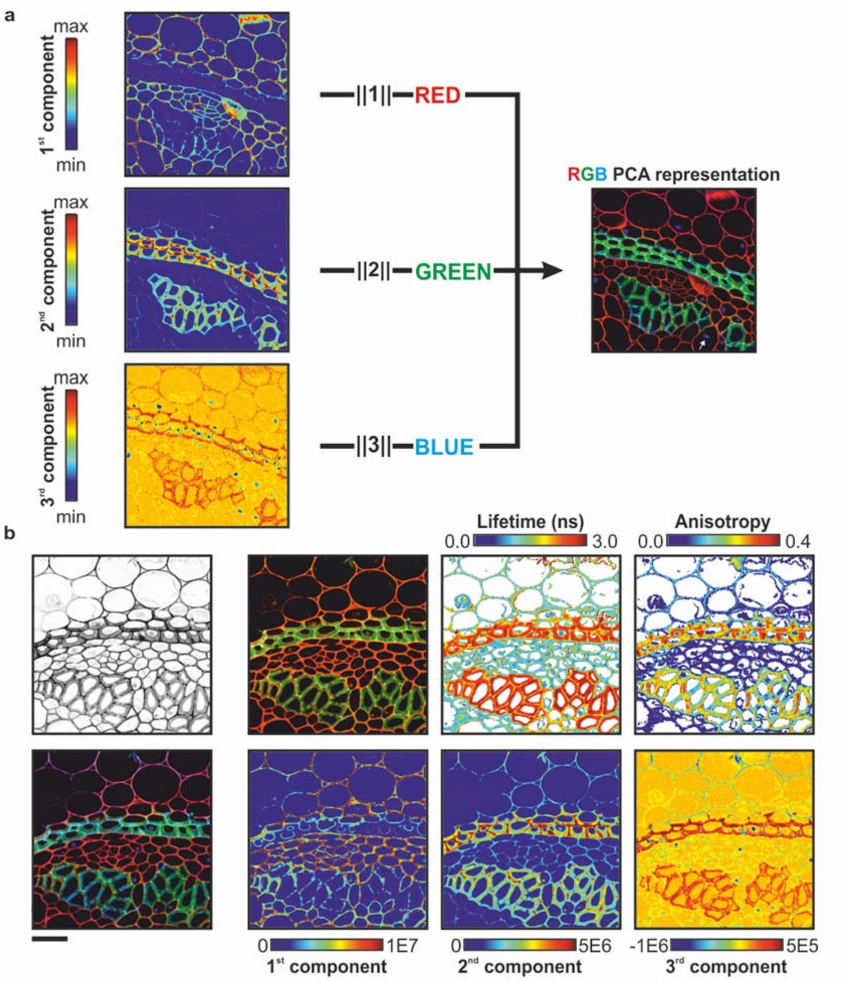

Figure S6 | Multivariate analysis of HDIM. a) Schematic representation of the generation of RGB composite of principal components. Principal components can be negative and, therefore, for simplicity, we present their absolute values. **b**) These panels are the same representations described in **Fig. 4** but computed to a second field of view to illustrate the robustness of the projections of calibrated dataset.

Supplementary Methods

Calibration procedure

A time-resolved spectropolarimeter is at the core of the HDIM platform. The system can be used uncalibrated and provide relative values rather than absolute measurements of polarization anisotropy, spectra and fluorescence lifetime. However, aiming to standardize each experiment, we have established a simple calibration of the temporal, polarization and spectral characteristics of the microscope:

- *Time calibration*: the TCSPC electronics is calibrated by the manufacturer and the only care that should be taken is the registration of time-bins with the laser pulse that is usually done at installation. However, to provide a reference value for each experimental session, we acquire one image of the common fluorescent acrylic plastics by Chroma Technologies.
- *Anisotropy calibration*: Anisotropy is computed with the following relation, where $I_{\parallel}$ and $I_{\perp}$are the photons counted by the detectors aligned parallel or orthogonal relative to the linear polarization of the laser:

  $$r(t) = \frac{I_{\parallel}(t) - GI_{\perp}(t)}{I_{\parallel}(t) + zGI_{\perp}(t)}$$

  G is a calibration factor commonly used to compensate for the depolarization of light caused by the objective and other optical elements. G was measured by imposing an anisotropy value identical to zero in all detection channels when measuring light emitted by a battery-operated white light emitting diode (LED) scattered by a frosted glass and positioned at the back aperture of the condenser lens.

  z is a depolarization value that depends on the numerical aperture of the objective and that can assume values between 1 (high NA) and 2 (collimated beam) (3). In this work, *z* is set equal to 2 - as in most of the literature - aiming to streamline the calibration process. The measured anisotropies will be slightly different from the absolute values but highly reproducible when using the same objective.

- *Spectral calibration*: spectral calibration was achieved by imaging the reflection of several laser lines with which any confocal microscope is equipped. The analyser of the confocal microscope was introduced into the optical path at the magic angle during this calibration in order to guarantee sufficient light on both polarization channels

Data analysis

Measurements from a white LED and a laser comb (see calibration procedure) were used to assign each of the 2,048 ‘detection channels’ to a specific wavelength, arrival time and polarization state triplet (λ, t, r), providing: i) a bijective function between the physical detector space to a calibrated triplet of axes and ii) the G factor and iii) a multiplicative pixel-dependent factor to correct for uneven illumination of the sample. All HDIM datasets were masked accordingly to a minimum desired total number of photons and calibrated by linear interpolation prior to any further data analyses. The threshold of photon-counts was usually

set at 400 photons, at which level we can expect – at best – a coefficient of variation of 5% because of the underlying Poissonian process (4). We note that from the release R2012b our code is working significantly faster compared to earlier versions of Matlab: <5s (compared to ~1 minute) for the interpolation of a typical HDIM dataset. Data analysis was performed on a Dell Precision T5500 equipped with an Intel Xeon X5647 operating at 2.93GHz, 24GB RAM and Windows 7 Professional 64-bits.

The *HDIM-toolbox* is a suite of Matlab scripts (~6,000 lines of commented code) designed specifically to analyse this new type of data sets. After including HDIM-toolbox and its subfolders into Matlab paths, HDIM_CAL, HDIM_START and HDIM_CA can be used to calibrate, inspect and perform multivariate analysis on HDIM datasets, respectively. HDIM-toolbox includes several scripts, but the main functions are described here below.

The definition of structures useful for analysis is provided by *if_hdim_init*, which returns HDIM_CAL (a structure storing all calibration parameters), HDIM_PAR (a structure storing information about imaging parameters), HDIM_VIS (a structure storing look-up tables designed for enhanced visualization of HDIM datasets and masks), and HDIM_CST (a structure storing some essential constant definitions). *if_hdim_load* provides a basic interface to load data, currently compatible with MAT, or Becker&Hickl SDT files.

HDIM_START load and call if_hdim_overview which executes a series of projections to demo several functions that we provide. These include, *if_hdim_anisotropy* that generate an average steady-state anisotropy projection image, visualize the average spectrally resolved anisotropy spectrum and stready-state anisotropy images for each spectral bin. *if_hdim_lifetime* that provide an average fluorescence lifetime image and *if_hdim_spec2rgb* that converts a spectral image to an RGB composite either binning distinct spectral bins as detected, or weighted as per human eye sensitivity. *if_hdim_plot3d* can be used to visualize HDSS with a 3D plot, as an average of the field of view.

HDIM_PCA executes a sequence of scripts to perform multivariate data analysis. This script includes call to *if_hdim_pca_reshape* and *if_pca_compute* that reshape the HDIM matrices for fast computation executed by *if_pca_compute*. Once the loadings are estimated, *if_hdim_pca_apply* perform the projections of the HDIM dataset onto the principal components. Notably, *if_hdim_pca_reshape* can perform a number of data standardizations and dimensionality reduction before feeding pixel information (treated as multi-dimensional replicates for PCA). If activated, PCA is fed not the raw HDIM photon-counts, but physical quantities such as the anisotropy values r_0 and r_∞, photon-counts and phasor transforms for each spectral bin. We also provide scripts for visualization of multi-variate data analysis including *if_hdim_pca_show*, *if_hdim_pca_rgb2dab* to output pathology-like digital stains. We also provide two International Color Consortium (ICC) profiles for the RGB and CYMK projections.

Together with HDIM-toolbox we provide data and calibration files to familiarize with the software. We also provide the code to generate the calibration file. HDIM_CAL execute a sequence of the most relevant script used to calibrate the system. *if_hdim_cal_files* and *if_hdim_calibration_gui* implement a minimal graphic user interface to select files used for calibration and to input essential parameters. Calibration of the spectral response of HDIM and of sensitivity of the two polarization-dependent channels (the G-factor) are performed by *if_hdim_cal_spectral* and *if_hdim_cal_anisotropy*, respectively. *if_hdim_cal_apply*, called by

HDIM_START and HDIM_CAL, interpolates the HDIM dataset to transform it into absolute coordinates of polarization, microtimes (lifetimes) and wavelengths.

Statistical analysis

Aiming to assess variations in biochemical/photophysical resolution in fluorescence microscopy experimentally, we presented results of statistical analysis in **Fig. 1g** and **Sup. Fig. 6**. The three-dimensional plots in **Fig. 1g** and **Supp. Fig. 6a-b** are generated by computing 3D histograms of either fluorescence lifetime/spectral peak/anisotropy or three principal component values for all pixels of all samples combined. The 3D histograms of the frequencies of these triplets of values were then normalized to the maximum occurrence and plotted by the three-dimensional rendering method of isosurfacing with a threshold value of 0.1. Therefore, the blue isosurfaces shown in **Fig. 1g** and **Supp. Fig. 6a-b** represent the location and scatter of the measurements with an intuitive graphical representation. For further quantitation, we detected the centre of each peak and plotted the line connecting each peak in red. The Euclidean distance between each sample was then logged. Then a profile of the distribution across each pair of peak along the direction defined by their respective connecting lines was computed and fitted with to gaussians. The standard deviation of the gaussian was then used to determine the scatter of the measurements. Supp. Note 2 and Supp. Fig. 1 illustrate this procedure for a single pair of samples with a two-dimensional measurement. Having logged a collection of all distances and scatter between all possible pairs of peaks, we then generated a list of separability values (S) in analogy to the seminal paper punlished by Koellner and Wolfrum (1992)(5) on fluorescence lifetime detection.

$$S_{ij} = \frac{\|peak_i - peak_j\|}{\sqrt{\sigma_i^2 + \sigma_j^2}}$$

Using the Student’s t-distribution with one degree of freedom, we evaluated the probability for each pair to be different and plotted a histogram of all these values inf **Supp. Fig. 6c-e**. The average separability values are plotted in **Supp. Fig. 6f-g**.

Supplementary Note 1 | Nomenclature

Hyperspectral microscopy is commonly defined as a technique that detects spectra within each pixel of an image in contrast to multi-colour or spectral systems that acquires images either at limited spectral bands or with sequential exposures of the sample. With a multi-dimensional microscope, we often refer to systems capable of 5D imaging, including 3D spatial imaging in time, with multiple colours. We realize that both the terms “hyperspectral” and “multi-dimensional” are insufficient to characterize unambiguously the technique we have established. Therefore, we refer to the simultaneous detection of polarization, colour and arrival times of photons as “hyper dimensional” to imply the acquisition of extra dimensions (diverse photophysical properties) relative to other techniques and, at the same time, to imply the clear relation to hyperspectral techniques. Therefore, we introduce the non-ambiguous acronym of HDIM to differentiate our technique from the many other existing imaging techniques that provide lower information. With hyper dimensional spectral signature (HDSS), we define a three-dimensional matrix representing the normalized photophysical signature of a sample characterized by its fluorescence emission decays at several contiguous spectral bands and for the two polarization states.

Supplementary Note 2 | Theory of Hyper-dimensional Imaging Microscopy

The theoretical analysis of multi-channel multi-parametric detection is rather complex and relies on the description of the Fisher information, *i.e.* the information that can be measured on a specific parameter (*x*) by means of a set of measurements ($\overrightarrow{HDSS}$). We have published the mathematical foundation of HDIM, demonstrating the theoretical grounds for maximization of the biochemical resolving power of a fluorescence microscope elsewhere(6).

Briefly, the *photon partitioning theorem* states that when we increase the number of detection channels to analyse fluorescence, we obtain higher or equal information on the sample optical properties. We derived serval mathematical corollaries defining when a photon partition is non-trivial (information strictly increases) and clarifying how this increase in Fisher information can be balanced with costs and photon-losses when engineering a system. Finally, we show how non-trivial partitioning of photons with higher channel density increases the physico-chemical (or biochemical) resolution of a detection system.

Here, we rather present a geometrical interpretation of the above statements that should appeal more to the interested but non-specialist reader. Supplementary Figure 1 illustrates the case where two independent features (*e.g.*, colour and anisotropy) of two different physicochemical environments are measured with some uncertainty. By simple geometrical considerations, it is possible to appreciate that the distance between the two environments in a bi-dimensional representation (*e.g.*, colour *versus* anisotropy) is larger or equal then the one that could be measured in a one-dimensional spectrum. Furthermore, again by simple geometrical considerations it is possible to show that the resolving power of the instrument increases ($R_C = \sqrt{R_A^2 + R_B^2}$) with a multi-dimensional detection scheme.

Supplemental references

1. Esposito, A., A. N. Bader, S. C. Schlachter, D. J. van den Heuvel, G. S. Schierle, A. R. Venkitaraman, C. F. Kaminski, and H. C. Gerritsen. 2011. Design and application of a confocal microscope for spectrally resolved anisotropy imaging. Opt. Express 19(3):2546-2555.
2. Lakowicz, J. R. 1999. Principles of Fluorescence Spectroscopy. Kluwer Academic/Plenum Publishers, New York.
3. Suhling, K., J. Levitt, and P. H. Chung. 2014. Time-resolved fluorescence anisotropy imaging. Methods Mol Biol 1076:503-519.
4. Gerritsen, H. C., M. A. Asselbergs, A. V. Agronskaia, and W. G. Van Sark. 2002. Fluorescence lifetime imaging in scanning microscopes: acquisition speed, photon economy and lifetime resolution. J. Microsc. 206(3):218-224.
5. Kollner, M., and J. Wolfrum. 1992. How many photons are necessary for fluorescence-lifetime measurements. Chem. Phys. Lett. 200(1-2):199-204.
6. Esposito, A., M. Popleteeva, and A. R. Venkitaraman. 2013. Maximizing the biochemical resolving power of fluorescence microscopy. PLoS One 8(10):e77392.
